# Statin Utilization Among Individuals Infected With Hepatitis C Virus: A Retrospective Cohort Study

**DOI:** 10.7759/cureus.36049

**Published:** 2023-03-12

**Authors:** Spencer R Goble, Philippe Nyembo, Holly Rodin, George Konstantinides, Jesse Powell, Amanda J Noska

**Affiliations:** 1 Department of Internal Medicine, Hennepin Healthcare, Minneapolis, USA; 2 Hennepin Healthcare Research Institute, Hennepin Healthcare, Minneapolis, USA; 3 Department of Pharmacology, Hennepin Healthcare, Minneapolis, USA; 4 Department of Pharmacology, University of Minnesota College of Pharmacy, Minneapolis, USA; 5 Department of Gastroenterology and Hepatology, Hennepin Healthcare, Minneapolis, USA; 6 Department of Infectious Diseases, Hennepin Healthcare, Minneapolis, USA; 7 Department of Infectious Diseases, University of Minnesota School of Medicine, Minneapolis, USA

**Keywords:** cirrhosis, primary prevention, coronary artery disease, statins, hepatitis c virus

## Abstract

Introduction and Objectives

Statin use for primary prevention of coronary artery disease (CAD) has historically been limited in patients with chronic liver disease due to concerns for increased adverse events with statin use in this population. We aimed to quantify the underutilization of statins among individuals with a history of HCV infection in a community health system to understand the clinical implications of statin underutilization in a diverse, generalizable population of patients infected with HCV.

Materials and Methods

We performed a single-center retrospective study of individuals with a history of HCV infection aged 40-75 years from 2019-2021. Statin eligibility was determined using the 2019 American College of Cardiology/American Heart Association (ACC/AHA) guidelines with the 2013 Pooled Cohort Equation used to determine atherosclerotic cardiovascular disease (ASCVD) risk. Baseline characteristics and adverse events of statin and non-statin users were compared, and factors associated with statin use were determined using multivariable logistical regression.

Results

Based on 2019 ACC/AHA guidelines, 752/1,077 (69.8%) subjects had an indication for a statin, 280/752 (37.2%) of which were treated with a statin. Cirrhosis was independently associated with statin underutilization. Diabetes, anti-hypertensive use, and Black race were all independently associated with statin use in subjects with an indication for therapy. Statin use was not associated with adverse events.

Conclusions

Statins were underutilized and well tolerated in the cohort of individuals with a history of HCV infection. This high-risk population would benefit from increased CAD screening and utilization of statins for the primary prevention of CAD.

## Introduction

Hepatitis C virus (HCV) infection is an independent risk factor for coronary artery disease (CAD), with recently reported odds of CAD among those with a history of HCV infection ranging from 1.3 to 4.2 [1-5]. With the emergence of direct-acting antivirals (DAA) leading to high cure rates of HCV and improving overall mortality in individuals infected with HCV, an increased emphasis on primary prevention of cardiovascular disease is warranted to address the shifting needs of this population at high risk for CAD [6-8]. Statins are frequently used to reduce the risk of CAD in a wide array of patients. Current American College of Cardiology and American Heart Association (ACC/AHA) guidelines recommend the use of statins for CAD primary prevention in patients 40-75 years of age with diabetes or calculated 10-year atherosclerotic cardiovascular disease (ASCVD) risk ≥ 7.5% (using the ACC/AHA 2013 Pooled Cohort Equation), or for individuals 20-75 years of age with low-density lipoprotein (LDL) ≥ 190 mg/dL [9]. Statins may also reduce non-cardiovascular complications of chronic liver disease. Indeed, multiple retrospective and prospective studies have demonstrated that statins can not only be used safely among patients with advanced liver disease, but statin use in patients with chronic liver disease is associated with decreased rates of fibrosis progression, hepatocellular carcinoma, and even mortality [10-15].

Despite the increased risk for vascular disease noted in patients with a history of HCV infection and the possible liver-specific benefits of statin therapy in chronic liver disease, statins are underutilized in this patient population [1,16-18]. Concerns about possible side effects likely play a role in underutilization; however, serious side effects such as worsening liver failure and rhabdomyolysis have been shown to be exceedingly rare in patients with liver disease except in decompensated cirrhosis [15,19-23]. The low risk and potential benefits of statins in many patients with a history of HCV infection provide compelling reasons to investigate statin utilization in these patients. Published literature to quantify underutilization has primarily assessed male veteran patients [1,16]. The limited variability in the previously assessed populations limits the generalizability of the findings. We aimed to quantify statin underutilization based on calculated 10-year ASCVD event risk in patients with a history of HCV infection in a community hospital setting to assess a more diverse, heterogenous, generalizable population. This article was previously presented as a meeting abstract at the 2022 American College of Gastroenterology Annual Scientific Meeting on October 24, 2022.

## Materials and methods

Study design and patients

We conducted a retrospective analysis from 1/1/2019-12/31/2021 of patients with a history of HCV infection at a large urban, safety-net community hospital. Individuals between the ages of 40-75 years with a history of HCV infection defined as the presence of HCV antibody (anti-HCV) or a positive qualitative or quantitative ribonucleic acid (RNA) test were included in the study. No distinction was made between active and resolved or treated HCV infection. To more accurately assess prescribing patterns, we excluded individuals who did not have an encounter within our health system from 1/1/2019-12/31/2021, as there would not have been an opportunity for a provider to evaluate them for appropriateness of statin therapy during the study period. Individuals who did not have adequate information to complete the 2013 Pooled Cohort Equation (age, sex, race, diabetes status, smoking status, antihypertensive treatment status, systolic blood pressure, total cholesterol, high-density lipoprotein) were excluded due to inability to assess if they met 2019 ACC/AHA guidelines for statin therapy as were those with an LDL value ≥ 190 due to ACC/AHA guidelines recommending against the use of the Pooled Cohort Equation in these individuals [1]. Those with chronic hepatitis B and human immunodeficiency virus (HIV) were excluded to avoid confounding variables. Individuals with baseline ASCVD were excluded as only utilization for primary prevention was being assessed. Baseline ASCVD was defined as a previous diagnosis of the acute coronary syndrome, myocardial infarction, angina (stable or unstable), cerebral infarction, transient ischemic attack, or peripheral artery disease. Regarding aspects of the Pooled Cohort Equation, antihypertensive treatment was defined as the presence of a prescription for any anti-hypertensive medication between 1/1/2019-12/31/2021. Systolic blood pressure was recorded using the median of the subjects’ three most recent recorded blood pressures before 1/1/2022 unless the patient had less than three measurements, in which case the most recent value before 1/1/2022 was used. For total cholesterol and high-density lipoprotein (HDL), the most recent values before 1/1/2022 were used.

The primary objective of this study was to determine statin utilization based on the calculated ASCVD risk score. The 2013 Pooled Cohort Equation was used to calculate the 10-year ASCVD risk for each included subject. Statin use was defined as the presence of a prescription for any statin between 1/1/2019-12/31/2021. To assess if liver function effected prescribing patterns, aspartate aminotransferase (AST), alanine aminotransferase (ALT), and platelet count were obtained for each subject, with the most recent values before 1/1/2022 used. Cirrhosis was defined as the documented clinical history of cirrhosis of any etiology or a Fibrosis-4 (FIB-4) score ≥ 3.25. The secondary objective of this study was to determine if statin use was associated with increased liver and muscle-specific adverse events. The incidences of ALT > 82 IU/L (reference range < 41 IU/L), AST > 80 IU/L (reference range 5-40 IU/L), new-onset ascites, and hepatic encephalopathy were recorded 1/1/2019-12/31/2021. The most recent ALT and AST values before 1/1/2019 were also recorded, and those with an ALT > 61.5 IU/L and/or an AST > 60 IU/L were excluded from the analysis of elevated ALT and AST to avoid interpretation of chronic elevations as acute liver injuries. For the same three-year period, incidences of myopathy and creatinine kinase (CK) > 600 IU/L (reference range 39-308 IU/L) were recorded to assess for muscle-related adverse events.

Statistical analyses

Demographic and clinical characteristics were examined and compared between subjects prescribed statins and those not prescribed statins and between different ASCVD risk stratifications. Continuous variables are presented as means with standard deviations, and categorical variables are presented as proportions. The Student’s t-test was used to compare continuous variables with a normal distribution assumed. Fisher’s exact test was used to compare 2x2 proportions, and chi-square was used to assess all other proportion comparisons. Multivariable logistical regression was used to assess for independent factors associated with statin utilization with odds ratios and 95% confidence intervals presented for each variable. SAS Software Version 9.4 (The SAS Institute, Cary, NC) was utilized for statistical computations.

## Results

A total of 1,077 subjects were included in the cohort, 244 of which had cirrhosis. During the study period, 306 subjects were prescribed a statin, and 771 subjects were not prescribed a statin. The baseline characteristics of statin and non-statin users are summarized and compared in Table 1. Statin users and non-statin users differed significantly in age, race, body mass index (BMI), smoking status, and ASCVD risk score. Cirrhosis was less prevalent in statin users than non-statin users, but this difference was not statistically significant.

**Table 1:** Characteristics of patients with a history of hepatitis C virus infection treated and not treated with statins. Note: Continuous variables are reported as means with standard deviations. Categorical variables are presented as proportions. The means of continuous variables were compared with the Student’s t-test. Proportions were compared using Fisher’s exact test with the degrees of freedom for each corresponding P-value = 1. ASCVD: Atherosclerotic cardiovascular disease; SD: Standard deviation. *p < 0.05, **p < 0.001

Variable	Statin (n = 306)	No statin (n = 771)	P-value
Age (years), mean (SD)**	59.5 (6.5)	55.9 (7.8)	< 0.001
Female sex*	31.0%	39.7%	0.008
Race			
Black**	56.2%	44.6%	< 0.001
White/Non-Hispanic**	27.5%	39.0%	< 0.001
Hispanic	6.5%	6.1%	0.781
Other	9.8%	10.2%	0.911
Total cholesterol (mg/dL), mean (SD)	168.0 (42.6)	169.3 (37.1)	0.626
Low-density lipoprotein cholesterol (mg/dL), mean (SD)	78.5 (42.7)	75.4 (40.0)	0.263
On anti-hypertensive therapy**	80.7%	51.0%	< 0.001
Diabetes**	58.8%	18.8%	< 0.001
Body mass index (kg/m^2^), mean (SD)**	30.5 (7.2)	28.4 (6.6)	< 0.001
Smoking status			
Current*	50.0%	60.8%	0.001
Former*	27.5%	19.7%	0.007
Cirrhosis	19.0%	24.1%	0.076
Treatment with HCV direct-acting antiviral at any time 2019-2021	30.7%	32.0%	0.717
10-year ASCVD risk score, mean (SD)**	0.205 (0.126)	0.115 (0.095)	< 0.001

**Figure 1:**
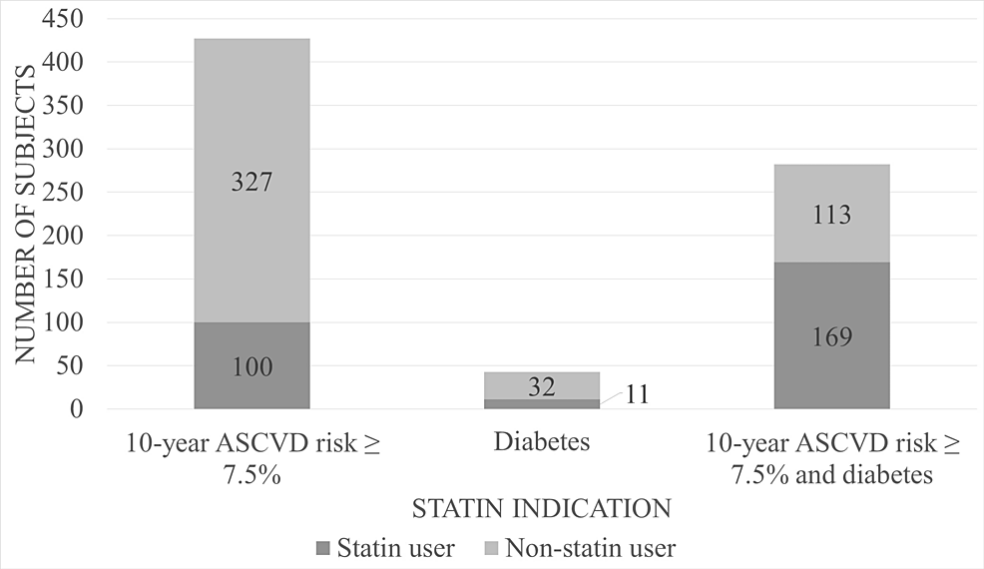
Statin utilization by 2019 American College of Cardiology/American Heart Association guideline-based indication for primary prevention of cardiovascular disease in the cohort of individuals with a history of hepatitis C virus infection. ASCVD: Atherosclerotic cardiovascular disease.

**Figure 2:**
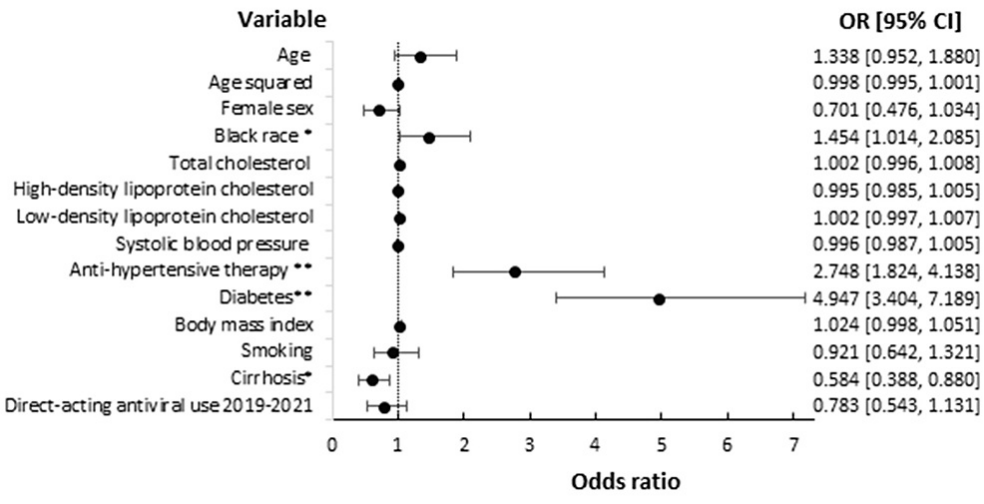
Multivariable logistic regression model demonstrating predictors of utilization in individuals with an indication for statin therapy. CI: Confidence interval; OR: Odds ratio. *p < 0.05, **p < 0.001.

Statin utilization increased as ASCVD risk increased and ranged from 8.0% in individuals with a 10-year risk of < 5% to 53.6% in individuals with a risk of ≥ 20%. Based on 2019 ACC/AHA guidelines, 752/1,077 (69.8%) of subjects had an indication for statin therapy. The number of subjects meeting each indication and the proportion treated with a statin is illustrated in Figure 1. In total, 280/752 (37.2%) individuals with an indication for statin therapy were prescribed a statin. When patients with cirrhosis were excluded, a total of 223/571 (39.1%) subjects with an indication for statin use per guidelines were treated with a statin. Utilization differed between the FIB-4 scores ranging from < 1.45, 1.45-3.24, and ≥ 3.25, with statins prescribed in 144/347 (41.5%), 91/272 (33.5%), and 27/95 (28.4%) of individuals with an indication for their use, respectively (p < 0.05). In a multivariable logistic regression model, Black race, diabetes, and anti-hypertensive use were all independently associated with statin use in individuals with an indication for therapy, and cirrhosis was associated with decreased use (Figure 2). While age, smoking status, systolic blood pressure, HDL, and BMI were all found to differ significantly between statin users and non-statin users, none were found to be independently associated with utilization.

In total, 59 adverse events were recorded among 58/1,077 (5.39%) different subjects. Statin use was not associated with increased adverse events in subjects with cirrhosis and those without cirrhosis. Individuals with cirrhosis were more likely to have an adverse event (p < 0.001). Table 2 summarizes the rate of each adverse event by statin use and the presence of cirrhosis. No episodes of hepatic encephalopathy were recorded.

**Table 2:** Adverse event rates by statin use and cirrhosis status. ¥ Analysis excluded individuals who had baseline elevations in AST and/or ALT defined as AST > 60 IU/L and/or ALT > 61.5 IU/L on the most recent measurement before 1/1/2019. Note: Proportions were compared using Fisher’s exact test. Degrees of freedom for each specified P-value = 1. ALT: Alanine aminotransferase; AST: Aspartate aminotransferase; CK: Creatine kinase; IU: International units

	Subjects without cirrhosis			Subjects with cirrhosis		
Adverse event	Statin use (n=248)	No statin use (n=585)	P-value	Statin use (n=58)	No statin use (n=186)	P-value
Myopathy	0/248 (0.00%)	1/585 (0.17%)	1.000	0/58 (0.00%)	0/186 (0.00%)	1.000
CK > 600 IU/L	1/248 (0.40%)	5/585 (0.85%)	0.675	0/58 (0.00%)	4/186 (2.15%)	0.575
Ascites	0/248 (0.00%)	1/585 (0.17%)	1.000	1/58 (1.72%)	4/186 (2.15%)	1.000
AST > 80 IU/L and/or ALT > 82 IU/L ¥	5/228 (2.19%)	15/506 (2.96%)	0.633	4/36 (11.11%)	18/119 (15.13%)	0.786

## Discussion

This data suggests that statins are underutilized for the primary prevention of CAD in individuals with a history of HCV infection. Previous studies demonstrated underutilization of statins in male veterans with a history of HCV infection, and this study demonstrates similar results among a more diverse, heterogenous, and thus more generalizable population [1]. A large number of subjects (69.8%) met the criteria for statin use based on 2019 ACC/AHA guidelines. While statin utilization did appropriately increase as ASCVD risk increased, even in very high-risk individuals (10-year risk ≥ 20%), statins were only prescribed in 53.6% of eligible subjects. Only 55.4% of subjects with diabetes were prescribed statins. In the United States general population, statin eligibility has been estimated to be 39.8%, and utilization for individuals with 10-year risk ≥ 20% and for those with diabetes have been calculated to be 60.6% and 73.6%, respectively [24].

Active HCV infection (RNA positive) has been shown to increase the risk of CAD [5]. While the degree of increased risk does not appear to be as significant, anti-HCV seropositivity itself is an independent and well-documented risk factor for CAD, suggesting that a history of HCV infection increases the risk of CAD even when active infection is not present [3-5,25]. The 2013 Pooled Cohort Equation has been shown to underestimate the risk of CAD in patients with a history of HCV infection with ASCVD risk scores ≥ 7.5% [1]. The increased risk of CAD in individuals seropositive for anti-HCV combined with the underutilization of statins for primary prevention creates a significant opportunity to improve outcomes with changes in clinical practice. Statins have demonstrated efficacy in reducing CAD in the general population [9]. Individuals with a history of HCV infection have largely been excluded from studies evaluating statin efficacy, but limited data are available that suggest statins reduce the incidence of acute myocardial infarction in individuals with active HCV [26]. Statins are underutilized among persons with a history of HCV infection based on our data and the available safety data for statin use in this population, especially when the demonstrated cardiovascular benefits of use among persons with chronic liver disease and the general population are considered. Further prospective assessment of the impact of statin use on the spectrum of ASCVD in individuals with a history of HCV infection is needed to fully understand the impact of statin use in this population.

Concerns for increased side effects in patients with liver disease may have been responsible for the underutilization of statins in this cohort. This theory was supported by our data which showed decreased utilization of statins in those who qualified for one as the FIB-4 score increased and by the independent association of cirrhosis with statin underutilization. Statins, particularly at higher doses, have been associated with acute increases in AST and ALT, myopathy, and rhabdomyolysis [27,28]. Increased rates of rhabdomyolysis have been noted in patients with advanced (bilirubin > 5 mg/dL) and decompensated cirrhosis, justifying caution with statins in advanced liver disease [22]. However, large-scale retrospective studies and randomized control studies have shown similar rates of acute liver injuries and muscle-related adverse events in patients with the compensated chronic liver disease taking statins compared to those not taking statins [15,19,21,29]. Consistent with previous studies, we found no increase in adverse events in patients with chronic liver disease and taking statins in this analysis. Our results suggest that education directed towards providers on the safety of statins in those with chronic, stable liver disease is warranted to improve utilization in this high-risk population.

While our results suggested that statin use decreases with more advanced liver disease, utilization was relatively low even in those without a diagnosis of cirrhosis or a FIB-4 score suggestive of advanced liver disease. Considering recent literature that has associated statin use with decreased rates of fibrosis progression, decreased incidence of hepatocellular carcinoma, and possibly even a decrease in mortality, this creates another potential opportunity for clinical improvement [12,14]. Further prospective research is needed to confirm these benefits, but the available retrospective data and some limited current prospective data are promising.

Diabetes and anti-hypertensive use were both independently associated with statin use in those with an indication. Cholesterol values (HDL, LDL, and total cholesterol), systolic blood pressure, age, and smoking were not. These findings suggest frequent identification of diabetes and hypertension (that requires anti-hypertensive treatment) as significant risk factors for CAD and also imply that clinicians are less reliant on solitary blood pressure measurements or laboratory evidence of CAD risk in the form of hypercholesterolemia for determining statin appropriateness in individuals with a history of HCV infection. Anti-hypertensive therapy may also be associated with greater engagement in care and willingness to be prescribed a statin. Similarly, the smoking status may be confounded by disengagement in care and other social stressors, which make CAD primary prevention less of a priority. We suspect the increased utilization of statins in Black individuals with a history of HCV infection compared to non-Black individuals reflects clinical use of the Pooled Cohort Equation, which includes the Black race as a CAD risk factor. The lack of association with age despite its associated risk of CAD is possibly due to less perceived benefit at more advanced ages.

Several limitations of this study warrant discussion. No distinction was made between active HCV infection and resolved or treated HCV infection, which prevents conclusions from being drawn on the impact active versus resolved disease may have on prescribing patterns. Given the increased risk of CAD noted in those with active disease compared to individuals who are solely anti-HCV seropositive, this difference warrants investigation [5]. Similarly, potential differences in the safety of statins based on HCV disease activity cannot be commented on based on these results. This is a single-center study with a relatively low sample size, which does reduce some of its generalizability, although the diverse population cared for within our health system and included in this study is certainly a balancing strength. The retrospective nature of this study limits the interpretation of clinical factors associated with adverse events and several important clinical factors to consider when evaluating for adverse event risk, such as Child-Pugh class, the specific statin prescribed, and actual medication fill rates were not included due to the limitations of the sample size and available data. We also acknowledge that many clinical factors that could influence the appropriateness of statin therapy are not captured in this work. This study was performed within a safety net community hospital with a patient population that experiences a multitude of psychosocial stressors. CAD risk is influenced by social determinants of health, and we think the inclusion of this population is an overall strength as it contributes to the existing literature on CAD risk and primary prevention in vulnerable populations [30]. However, the social stressors faced by this population may influence statin prescribing patterns due to prioritization of social issues and barriers to care which may limit the ability of patients to follow up, and these factors were not accounted for in the study design. A prospective study design that can more accurately account for outcomes based on follow-up measures is needed to confirm some of the findings of our study. We did not include a matched cohort of individuals without a history of HCV infection which would have allowed for comparisons within our system to better isolate the true impact of HCV infection on statin use.

## Conclusions

This study found that statins are underutilized in patients with a history of HCV infection and suggests the need for increased utilization of statins for primary prevention of CAD among this high-risk population. Concerns for safety issues have limited the use of statins in chronic liver disease, despite considerable evidence showing the relative safety of statin use in compensated chronic liver disease. Further research is needed to determine the precise impact statins have on the development of CAD in those with a history of HCV infection. However, based on data from the general population and from those with active HCV, significant benefits in morbidity and mortality could potentially be realized with greater statin utilization for primary prevention among persons with a history of HCV infection. Our study suggests that statins are generally safe and well-tolerated in this population.
